# Prevalence of human immunodeficiency virus and Hepatitis (B & C) among drug users in a tertiary care public hospital

**DOI:** 10.12669/pjms.35.2.500

**Published:** 2019

**Authors:** Aftab Asif, Sumaira Ayub, Afreen Komal, Shahbaz Noor, Umer Jalal

**Affiliations:** 1 *Prof. Dr. Aftab Asif, MBBS, MRCPsych. Chairperson, Academic Department of Psychiatry and Behavioral Sciences, King Edward Medical University / Mayo Hospital, Lahore, Pakistan*; 2 *Sumaira Ayub, PhD Scholar. Research Assistant, Academic Department of Psychiatry and Behavioral Sciences, King Edward Medical University / Mayo Hospital, Lahore, Pakistan*; 3 *Afreen Komal, PhD Scholar. Lecturer, University of Management and Technology, Lahore, Pakistan*; 4 *Shahbaz Noor, MBBS, MRC Psych. Assistant Professor, Academic Department of Psychiatry and Behavioral Sciences, King Edward Medical University / Mayo Hospital, Lahore, Pakistan*; 5 *Umer Jalal, MBBS. Academic Department of Psychiatry and Behavioral Sciences, King Edward Medical University / Mayo Hospital, Lahore, Pakistan*

**Keywords:** Prevalence, Drug users, Human immunodeficiency virus, HIV, Hepatitis B, Hepatitis C

## Abstract

**Objective:**

To find out the prevalence of human immunodeficiency virus (HIV) and Hepatitis (B & C) among the drug users in a tertiary care public hospital.

**Methods:**

The study was conducted at addiction ward of Mayo Hospital Lahore. A total of 453 drug users were admitted in drug addiction ward from 1^st^ of August 2016 to 31^st^ of July 2017. Their history was taken using self-constructed case history form and they were screened for HIV and hepatitis (B & C). Additionally three types of screening tests including Unigold, Determine and Bioline were used for HIV screening.

**Results:**

Of 402 (100%) drug users, 394 (98%) were male and 8 (2%) were female. Their mean age was 32.2 (8.8) years. Overall prevalence of HIV virus was 21.1%, Hepatitis C 34.3% and Hepatitis B 3.2% in drug users. Among HIV positive drug users, 84.7% drug users took drugs through injections as compared to 15.3% who took drugs orally. Among HCV positive drug users, 68.9% drug users took drugs through injections as compared to 31.1% oral drug users. Relapse rate of drug use among all drug users was also very high as 83.3%. Of these drug users, 47.2% had previously sought treatment while other 52.8% never sought any treatment. Family history of drug abuse indicated that 32.2% drug users had family members who were also drug users. Moreover, about 11.4% drug users had previous history of blood transfusion.

**Conclusion:**

HIV and hepatitis B & C were prevalent among drug users specially those who took drugs through injections. Relapse rate was significantly high and history of drug use in family may also predispose an individual towards becoming a drug addict.

## INTRODUCTION

Blood-borne viral infections are a major health problem in intravenous drug users. Drug and alcohol use prone the individuals at high risk of viral hepatitis and HIV. Hepatitis B virus (HBV) and hepatitis C virus (HCV) are leading health threats among injecting drug users. Intravenous drug users suffer with multiple health conditions including mental illness and HIV thus requiring care from multiple healthcare providers. Approximately 0.8–1.4 million people are living with HBV and 2.7–3.9 million people are living with HCV in the United States.^1^ These blood-borne viral infections have reached epidemic proportions in most states, disproportionately affecting rural communities in addition to young persons. Individuals who become infected with HBV and HCV are also at increased risk for other diseases i.e. HIV transmitted through intravenous drug use.^2^

In 2007, it was estimated that there were 15.9 million intravenous drug users worldwide, with three million living with HIV. Drug abuse and addiction have been intricately linked with HIV/AIDS since the beginning of the epidemic. The epidemic is expanding among intravenous drug users with the prevalence of 20% approximately. National surveillance data shows rates of infection in most major cities ranging from 15% to 50% among the estimated 1,50, 000 intravenous drug users in Pakistan.^3^

Viral hepatitis is very contagious and those people who have direct contact with equipment, objects, or surfaces contaminated with infected blood are at great risk of HCV and HBV which may lead to severe liver damage and even death after many years of infection.^4^

Studies have showed that intravenous drug users are more likely to have HCV infection. People become infected with the HCV through direct contact with the blood of infected person, by sharing contaminated needles and other drug injection.^5^ It is clear that micro-environmental and macro-environmental physical, social, economic, and even political factors are important in shaping risk behaviors for HIV and HCV acquirement among intravenous drug users.

Asian Epidemic Modelling (AEM) conducted a study in 2015 and it was found that in Pakistan the main cause of HIV transmission among injecting drug users is the use of contaminated injection equipment. The estimated number of injecting drug users was ranged from 104,804 to 420,000. It was estimated that overall prevalence of HIV was about 40% in several cities, including Faisalabad (52.5%), D.G. Khan (49.6%), Gujrat (46.2%), Karachi (42.2%) and Sargodha (40.6%) respectively.^6^

According to a survey by United Nation (UN) carried in Pakistan it was highlighted that 48% of drug addicts who inject drugs in their bodies with syringes, are suffering from HIV. The data was taken from fourteen cities including Karachi, Kasur and Bahawalpur. The survey report unearthed alarming facts that 24 thousand drug addicts in Karachi were tested to have positive HIV. Furthermore, 50% samples in Kasur, and 25% in Bahawalpur showed the same results.^7^ In a survey conducted by Association of People Living with HIV on the sample of 545 who used drugs and were infected with HIV. Despite of high prevalence of HIV in the drug abusers, about 298(54.7%) were infected with HIV while 23(4.2%) reported being on ART (anti-retroviral therapy).^8^

In United States and in Europe, HCV is more prevalent among intravenous drug users. Among intravenous drug users 50-90 percent are infected with HCV.^9^ Like many other developing countries, Pakistan is also facing the elevated risk of HIV and Hepatitis which may be due to poverty, poor quality of education, gender based discrimination, lack of awareness about HIV transmission and the stigma that prohibits people with risk behaviors from seeking HIV testing or disclosing their HIV positive status.^10^

This study aims to ascertain prevalence of Hepatitis (B & C) and HIV among drug users and which socio-demographic variables incorporate in high prevalence rate.

## METHODS

This study was conducted at Drug Addiction center of Department of Psychiatry and Behavioral Sciences, Mayo Hospital Lahore after approval from Ethical Review Board of the institution. A total of 453 drug users were admitted in drug addiction ward from 1^st^ of August 2016 to 31^st^ of July 2017. Their case history was taken and they were screened for HIV and Hepatitis (B & C). Of 453 drug users, 51 (11.3%) did not get screened and left hospital uninformed as LAMA (leave against medical advice); therefore their data was discarded. All the ethical considerations were taken into account. Study was approved by Ethical Review Board of King Edward Medical University. Patients’ consent was taken and they were assured of confidentiality of the information they provided. Two main sources were used to collect data:

Firstly, self-structured case history form was used to collect detail information regarding age, gender, residence (rural, urban), qualification, occupation, marital status, number of children (if married), siblings, birth order, monthly income, SES (socio-economic status), duration of drug abuse, source, route of drug administration, family history of drug abuse, family history of psychiatric illness, comorbid psychiatric illness, any other medical disease, HIV status, history of blood transfusion, problem with police, history of imprisonment, number of previous treatments, number of relapses and mode of discharge.

Secondly, every patient was tested for HIV and hepatitis (B & C) through initial screening test. Patients who had positive status on initial screening tests were referred to special testing clinic for further confirmation. For HIV screening, three types of screening tests were used further: Unigold, Determine and Bioline. Patient’s diagnosis of HIV positive was confirmed if two of these tests were positive. Patients who were HIV positive were provided with special care.

Data was analyzed by using SPSS 20.0 version. Demographic variables were assessed through descriptive statistics using frequency, percentages, mean and standard deviation, while cross tabulation analysis was used to compare the relationship between diseases (HIV, HBV, HCV) and mode of drug use.

## RESULTS

The total sample of 402 patients was included in the present study. There were 394 male (98%) and 8 females (2%) with mean age of 32.2±8.9 years. Majority of the participants 254 (63.2%) were married and 294 (73.1%) addicts had urban background. Almost 74 (18.4%) of the addicts were had primary; 104 (25.9%) Middle; 61 (15.2%) Matric and 138 (34.3%) were illiterate. Professionally, about 133 (33.1%) were laborer and 90 (22.4%) were private employees, however 68 (16.9%) were those who were not doing any job. About 343 (85.3%) of the participants belonged to lower class. Out of 402 patients, 85 (21.1%) drug users were infected with HIV, 13 (3.4%) with HBV and 138 (34.3%) were infected with HCV. Out of 85 drug users with HIV positive, 72 (84.7%) were those who took drugs intravenously through injection, while 13 (15.3%) drug user did not take drugs through injections. Results also indicates that out of 135 drug users with HCV positive, 93 (68.9%) were those who took drugs intravenously through injection, while 42(31.1%) drug users did not. From results, it is also revealed that among 13 (3.4%) drug users with HBV, 7(53.8%) were those who took drugs through injections while 6 (46.2%) were those who did not take drugs through injections.

We also found that 265 (58.5%) of sample population consisted of those who have been using drugs for five years or less while 82 (18.1%) have been using drugs for 11 to 15 years. Three hundred seventy eight (83.4%) drug users reported to have relapsed in previous years of drug abuse, while 75 (16.6%) did not experience relapse. Of 402 patients, only 214 (47.2%) had sought treatment previously. A total of 137 (30.2%) reported to have history of drug abuse in their families and 54 (11.9%) reported history of psychiatric illness in their families. It was revealed that 48 (11.9%) have history of blood transfusion. About 169 (42%) reported problems with police and 143 (35.6%) had history of imprisonment.

## DISCUSSION

We found that HIV, hepatitis B or C are prevalent among drug users. In a study conducted by Vicknasingam, Narayanan and Navaratnam (2009) to find prevalence of HCV in drug users. 552 drug users were surveyed and it was found that prevalence of HCV was 65.4% in overall sample but it was higher among intravenous drug users (67.1%) relative to non-intravenous drug users (30.8%). They also found that majority (65.9%) of participants were sharing intravenous equipment and about the same proportion (65.4%) being HCV positive, the risk of further transmission to new drug users was high.^11^

**Table-I:** Status of disease and route of drug administration among drug users with HIV, Hepatitis B and C.

Disease	Status of Disease	Total (n=402)	Route of Drug Administration	

			Intravenous	Non-intravenous
HIV	Positive	85 (21.1)	72 (84.7)	13 (15.3)
	Negative	317 (78.9)	90 (28.4)	227 (71.6)
Hepatitis B	Positive	13 (3.4)	7 (53.8)	6 (46.2)
	Negative	371 (96.6)	139 (37.5)	232 (62.5)
Hepatitis C	Positive	135 (34.3)	93 (68.9)	42 (31.1)
	Negative	262 (65.7)	65 (24.8)	197 (75.2)

(Percentage in parenthesis)

**Table-II:** Information related to drug use *(N*=402).

Variables	f (%)
***Years of drug abuse***	
Less than a year	11(2.4)
1-5 years	265 (58.5)
6-10 years	6 (1.3)
11-15 years	82 (18.1)
16-14 years	52(11.5)
More than 20 years	37(8.2)
***Pre Abused Drug***	
Cannabis	278 (59.4)
Alcohol	41 (8.8)
Heroin	33 (7.1)
Opium	50 (10.7)
White Crystal	22 (4.7)
***Currently Abused Drug***	
Cannabis	22 (4.7)
Heroin	124 (26.4)
Opium	26 (5.5)
White Crystal	136 (28.9)
Marijuana	37 (7.9)
Button	23 (4.9)
Benzodia	27 (5.8)
Alcohol	4 (0.8)
***Relapse rate***	
Relapse	378 (83.4)
No relapse	75 (16.6)
***Previous treatment***	
Treatment	214 (47.2)
No treatment	239 (52.8)
***Family history of drug abuse***	
Present	137 (30.2)
Absent	315 (69.5)
***Family history of psychiatric illness***	
Present	54 (11.9)
Absent	397(87.6)
***History of blood transfusion***	
Present	48(11.9)
Absent	354(88.1)
***Problem with police***	
Yes	169(42.0)
No	233(58.0)
***History of imprisonment***	
Yes	143(35.6)
No	259 (64.4)

It is highlighted that 85.3% drug addicts belonged to a low socio-economic status. Another study was conducted on 250 drug users registered at addiction center in Amritsar during the year 2002. It was found that 9.6% (24) were found to be HIV positive, 23.6% (59) were HBV positive while 14.8% (37) were HCV positive. Moreover majority of addicts were belonging to poor socio-economic strata of society which is also a finding of present study.^12^

There are certain factors which are responsible for transmission of HIV and Hepatitis B & C. It is suggested from present study that about 11.9% of sample had previous history of blood transfusion. Moreover in a study by Danta, Brown, Bhagani, Pybus, Sabin and Nelson(2007) it was found that HCV is transmitted through blood contact including unsafe injection practices in medical and non-medical (e.g. intravenous drug use and tattooing) contexts^13^. HIV infection is also associated with homelessness, previous sexually transmitted infection, frontloading, back loading and daily injection. However, for long-term injectors, HIV risk factors were: having shared syringe, having injected cocaine, reporting front/back loading and ever having been in prison.^17^

A total of 137 (30.2%) reported to have history of drug abuse in their families and 54 (11.9%) reported history of psychiatric illness. People with serious mental illness are at risk of blood-borne viral infections which are probably multifactorial and associated with low socioeconomic status, drug and alcohol abuse, ethnic origin, and sex.^18^

White et al. studied the prevalence of HCV and HIV infection and associated risk behaviors among injection drug users in two northern Mexican cities. In both cities HCV and HIV were found to be highly prevalent about 96% and 2.8% respectively. About 87.5% intravenous drug users reported to pass on their used injection equipment to others and 85.9% received used equipment from others. Frequent sharing practices indicated a high potential for continued transmission for both infections.^15^

A study reported that high frequency of injecting drugs on daily basis was associated with HCV infection among young intravenous drug users in Vancouver, Baltimore, and San Francisco. It was also estimated that for every two years the youth continue to inject, can double the risk of hepatitis c among intravenous drug users. Therefore those who have been using intravenous drugs for six or more years are 10 times more likely to be HCV positive. So greater number of years in intravenous drug use is linked with positive HCV.^15^

## Limitation of the study

The patients were only taken from a tertiary care public hospital, in order to diversify the sample and generalize the findings patients from private hospital should also be included. The female patients were negligible in the present study, a separate study on female patients should be conducted in order to see the prevalence of HIV, HBV and HCV. Further, equal sample of female should be included in the future study in order to conduct the comparative study.

## CONCLUSION

Hepatitis C was more prevalent among drug users especially in those who were taking drugs through injections. Further HIV was also found to be second prevalent infection, however the rate of hepatitis B was not much high. Relapse rate for the drug use was significantly high among the drug users and history of drug use in family may also predispose an individual towards becoming a drug addict.

## References

[1] World health Organization (2017). HIV/AIDS.

[2] American Society of Addiction Medicine HIV/AIDS &Hepatitis C.

[3] Hagan H, Thiede H, Des Jarlais DC (2005). HIV/hepatitis C virus co-infection in drug users:risk behavior and prevention. Aids.

[4] Bergenstrom A, Achakzai B, Furqan S, Haq M, Khan R, Saba M (2015). Drug-related HIV epidemic in Pakistan:a review of current situation and response and the way forward beyond 2015. Harm Reduction J.

[5] Crofts N, Azim T (2015). Harm reduction in Asia and the Pacific:an evolving public health response. Harm Reduction J.

[6] Association of People Living with HIV (APLHIV) (2014). National Study on Access of Drug Users to Treatment &Fundamental Human Rights in Pakistan.

[7] (2016). United Nations Office on Drugs and Crime (UNODC) (2016) 'World Drug Report.

[8] Mathers BM, Degenhardt L, Phillips B, Wiessing L, Hickman M, Strathdee SA (2008). Global epidemiology of injecting drug use and HIV among people who inject drugs:a systematic review. Lancet.

[9] Strathdee SA, Hallett TB, Bobrova N, Rhodes T, Booth R, Abdool R (2010). HIV and risk environment for injecting drug users:the past, present, and future. Lancet.

[10] Vicknasingam B, Narayanan S, Navaratnam V (2009). Prevalence rates and risk factors for hepatitis C among drug users not in treatment in Malaysia. Drug Alcohol Rev.

[11] Aggarwal Aruna (2003). 'Prevalence of HIV and Hepatitis B and C among Drug Addicts in the Holy City of Amritsar, India'. 9th European AIDS Conference (EACS) Warsaw, Poland.

[12] Danta M, Brown D, Bhagani S, Pybus OG, Sabin CA, Nelson M (2007). Recent epidemic of acute hepatitis C virus in HIV-positive men who have sex with men linked to high-risk sexual behaviours. Aids.

[13] Shrestha IL (2003). Seroprevalence of antibodies to hepatitis C virus among injecting drug users from Kathmandu. Kathmandu Univ Med J.

[14] Hahn JA, Page-Shafer K, Lum PJ, Ochoa K, Moss AR (2001). Hepatitis C virus infection and needle exchange use among young injection drug users in San Francisco. Hepatology.

[15] White EF, Garfein RS, Brouwer KC, Lozada R, Ramos R, Firestone-Cruz M (2007). Prevalence of hepatitis C virus and HIV infection among injection drug users in two Mexican cities bordering the US. Salud Pública de México.

[16] Folch C, Casabona J, Espelt A, Majó X, Meroño M, Gonzalez V (2016). High prevalence and incidence of HIV and HCV among new injecting drug users with a large proportion of migrants—is prevention failing?. Subst Use Misuse.

[17] Hughes E, Bassi S, Gilbody S, Bland M, Martin F (2016). Prevalence of HIV, hepatitis B, and hepatitis C in people with severe mental illness:a systematic review and meta-analysis. Lancet Psychiatry.

